# Second-Harmonic Generation in Suspended AlGaAs Waveguides: A Comparative Study

**DOI:** 10.3390/mi11020229

**Published:** 2020-02-23

**Authors:** Iännis Roland, Marco Ravaro, Stéphan Suffit, Pascal Filloux, Aristide Lemaître, Ivan Favero, Giuseppe Leo

**Affiliations:** 1 MPQ, Université de Paris & CNRS, 10 rue A. Domon et L. Duquet, 75013 Paris, France; iannis.roland@univ-paris-diderot.fr (I.R.); marco.ravaro@univ-paris-diderot.fr (M.R.); stephan.suffit@univ-paris-diderot.fr (S.S.); pascal.filloux@univ-paris-diderot.fr (P.F.); ivan.favero@univ-paris-diderot.fr (I.F.); 2 C2N, CNRS, Université Paris-Saclay, 10 boulevard T. Gobert, 91120 Palaiseau, France; aristide.lemaitre@c2n.upsaclay.fr

**Keywords:** second-harmonic generation, waveguide, AlGaAs

## Abstract

Due to adjustable modal birefringence, suspended AlGaAs optical waveguides with submicron transverse sections can support phase-matched frequency mixing in the whole material transparency range, even close to the material bandgap, by tuning the width-to-height ratio. Furthermore, their single-pass conversion efficiency is potentially huge, thanks to the extreme confinement of the interacting modes in the highly nonlinear and high-refractive-index core, with scattering losses lower than in selectively oxidized or quasi-phase-matched AlGaAs waveguides. Here we compare the performances of two types of suspended waveguides made of this material, designed for second-harmonic generation (SHG) in the telecom range: (a) a nanowire suspended in air by lateral tethers and (b) an ultrathin nanorib, made of a strip lying on a suspended membrane of the same material. Both devices have been fabricated from a 123 nm thick AlGaAs epitaxial layer and tested in terms of SHG efficiency, injection and propagation losses. Our results point out that the nanorib waveguide, which benefits from a far better mechanical robustness, performs comparably to the fully suspended nanowire and is well-suited for liquid sensing applications.

## 1. Introduction

Recent technological advances have allowed reducing the size of semiconductor photonic devices to the sub-micrometer scale, with a remarkable impact in several research domains like integrated optofluidics [1] and nonlinear photonics [2]. Because of the high-refractive-index contrast and subwavelength size, the normal field component can be very strong at the semiconductor–air interface. This makes nanophotonic devices very sensitive to the complex refractive index of the surrounding medium and thus promising candidates for chemical or biological sensing in liquid or gaseous environments with lab-on-chip integrated photonic sensors [3]. This is all the more true for resonators and waveguides operating in the mid-infrared, where many absorption resonances of important analytes occur [4]. For these reasons, suspended silicon structures operating in the linear regime have been recently proposed as an alternative to their silicon-on-insulator counterparts [5,6], where the SiO_2_ substrate exhibits nonnegligible losses around 2.8 µm and beyond 4 µm, while the transparency of silicon itself ends beyond 8.5 µm [7]. A further asset of nanoscale high-contrast photonics in respect to µm-sized devices is the combination of strong nonlinear light–matter interaction with higher flexibility in dispersion and mode coupling engineering [8].

In this context, Al_x_Ga_1-x_As is an attractive material for its high second-and third-order nonlinear coefficients (d_14_ ≈ 100 pm·V^−1^ [9], n_2_ ≈ 10^−17^ m^2^·W^−1^ [10]), well-established processing technology, direct bandgap (for x < 0.45) that increases with Al molar fraction x and its broad transparency spectral region ranging from near- to mid-IR. The exploitation of AlGaAs nonlinearity for frequency mixing was once challenging because of its optical isotropy, which hinders birefringent phase-matching (PM), and its optical losses associated with the implementation of quasi-PM in the near-IR. In the last two decades, however, efficient guided-wave frequency mixing has been reported, based on form birefringence [11,12], modal PM [13] and counterpropagating PM [14]. In each of those cases, the nonlinear waveguides relied on total internal reflection between an aluminum-poor AlGaAs core and aluminum-rich claddings with a relatively low refractive-index step (Δn ≈ 0.2), which was also the case for the demonstration of χ^(3)^ guided-wave devices [15]. 

In the last years, high-contrast AlGaAs nonlinear photonic structures have been reported at the nanoscale level, based on either selective oxidation of an AlAs substrate [16,17] or epitaxial liftoff followed by bonding on glass [18], for both second-harmonic generation (SHG) [16,17,18] and spontaneous parametric down-conversion (SPDC) [19]. Their higher refractive-index step (Δn ≈ 1.5) made them suitable for shallow etching fabrication, with a huge impact on integration up until the demonstration of the first χ^(2)^ metasurfaces [20,21]. Similar AlGaAs-on-oxide structures have also been demonstrated for waveguides and microresonators fabricated by wafer bonding, both in χ^(3)^ [22] and χ^(2)^ devices [23,24]. However, the potential of AlGaAs-on-oxide guided-wave devices is still affected by either the intrinsic limits of wafer bonding technology in terms of homogeneity and throughput or by the intrinsic scattering loss of devices based on native AlAs oxide [25,26]. 

Within this context, an alternative approach to high-contrast AlGaAs photonics was pioneered more than a decade ago with substrate-removed electrooptic modulators [27,28]; then, suspended microdisk resonators were used both in optomechanics [29] and nonlinear optics [30,31,32]. Finally, suspended nonlinear nanowires [33] and nanorib waveguides [34] have been reported, and a suspended nonlinear photonic integrated circuit has been demonstrated for both SHG and SPDC in a microdisk coupled with two distinct waveguides at ω and 2ω [35]. 

Both nanowire and nanorib waveguides naturally lend themselves to mode birefringence phase-matching with a few advantages over multilayered form birefringent waveguides: (a) the attainable modal birefringence is sufficient to compensate dispersion in the whole AlGaAs transparency range, even close to the gap; (b) the modal areas of the fields are extremely small and tightly confined within the GaAs core, resulting in high conversion efficiency; and (c) the absence of aluminum oxide layers and the smoothness of top and bottom surfaces, which is defined by epitaxial growth, result in low scattering losses. 

**Figure 1:**
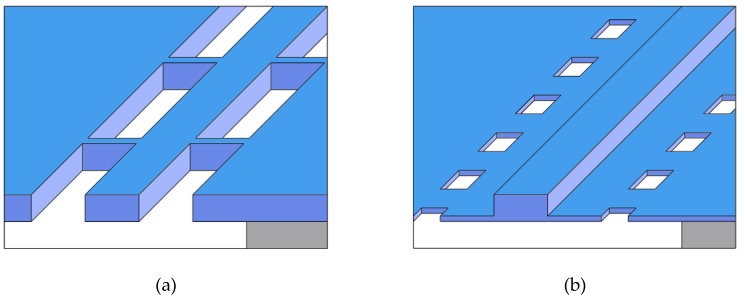
Suspended waveguide schemes: (**a**) nanowire anchored by tethers; (**b**) nanorib bounded by etch windows. Tethers and windows have no impact on optical propagation.

Here we compare the experimental performances and drawbacks of two different designs for AlGaAs suspended nonlinear waveguides (Figure 1): (a) a nanowire that recently allowed the demonstration of phase-matched SHG in both straight and snake-shaped configurations [33] and (b) a nanorib waveguide developed for frequency down-conversion towards the mid-IR range [34].

## 2. Materials and Methods

Both the above devices were processed from a planar AlGaAs heterostructure consisting of a 123 nm thick film of Al_0.19_Ga_0.81_As on top of a 4 μm thick Al_0.8_Ga_0.2_As layer, grown on a GaAs {001} substrate by molecular-beam epitaxy. 

**Figure 2:**
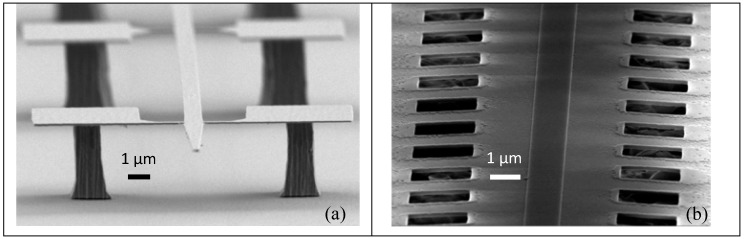
Scanning electron microscope (SEM) images of the suspended nanowire (**a**) and nanorib (**b**) waveguides.

Suspended nanowires 1 µm wide and 1 mm long (Figure 2a) were patterned along with their anchoring points by e-beam lithography followed by Ar/SiCl_4_-assisted inductively coupled plasma reactive-ion etching (ICP-RIE). The anchoring points were pairs of 100 nm wide and 1 μm long lateral tethers placed every 50 μm along the wire. A 1 mm wide, 100 µm deep mesa was then defined in the GaAs substrate by optical lithography and wet etching, giving access to the input and output ends for butt coupling. Finally, the Al_0.8_Ga_0.2_As layer was underetched with 1% HF solution at 4 °C for 6 minutes without stirring before sample CO_2_ critical point drying. 

Suspended nanorib waveguides (Figure 2b) were patterned by means of a two-step e-beam lithography plus ICP-RIE process: the former defined a 1 µm wide, 200 µm long and 80 nm thick rib in the Al_0.19_Ga_0.81_As layer, while the latter opened two lines of 2 µm × 2 µm square windows through the same layer, 2 µm away from the strip. The windows allowed wet isotropic underetching (10 min in 1% HF at room temperature with moderate stirring) of the underlying Al_0.8_Ga_0.2_As layer, which thus liberated a suspended 40 nm thick, 15 μm wide and 200 µm long Al_0.19_Ga_0.81_As membrane supporting the guiding rib. It is worth noticing that rib waveguides, due to intrinsic robustness, do not require critical point drying at the end of processing but can be simply flash dried (isopropanol evaporation on a hot plate at 270 °C). 

Both types of waveguides were terminated with inverted tapers designed for efficient input/output coupling at fundamental frequency ω and second-harmonic 2ω.

All devices were tested using two continuously tunable laser sources: aCW external cavity laser diode emitting between 1.5 and 1.6 µm and a single mode CW Ti:sapphire tunable between 0.7 and 1 µm. Both laser beams butt coupled at the input and the output with microlensed, single mode optical fibers. Linear and nonlinear spectra have been recorded by injecting and tuning the laser sources while detecting the outcoupled light either by an InGaAs or an Si photodiode.

## 3. Results

**Figure 3:**
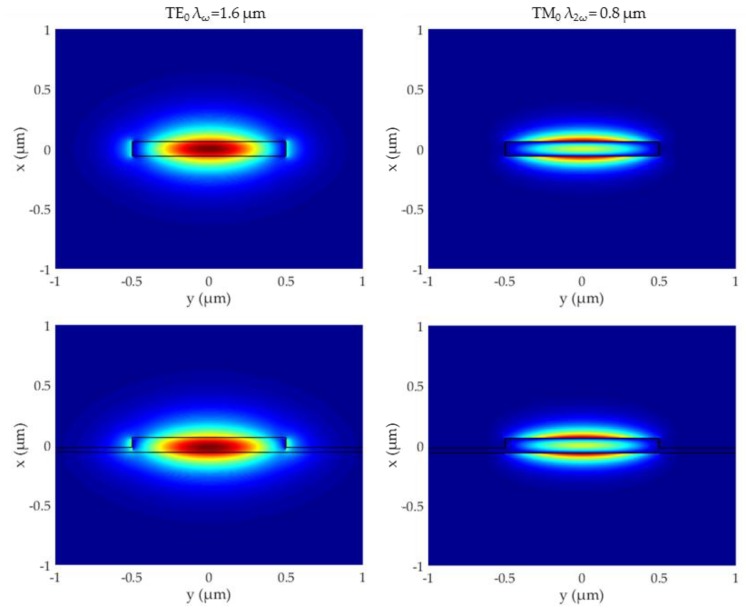
TE_00_ amplitude at ω (E_y_, left) and TM_00_ amplitude at 2ω (E_x_, right) in the suspended nanowire (top) and rib waveguide (bottom).

The transverse section of the waveguides was designed for Type-I phase-matched SHG from the TE00 mode at ω (λ ≈ 1.6 µm) to the TM00 mode at 2ω (λ ≈ 800 nm): (a) the thickness of the Al_0.19_Ga_0.81_As film was chosen so as to ensure strong modal birefringence while keeping the interacting modes well-confined; (b) the wire/rib width was then adjusted in order to precisely set the phase-matching wavelength [33]. The electric field amplitude profiles of both modes are shown in Figure 3. It can be observed that the 40 nm thick membrane does not significantly affect the lateral confinement for both modes. Accordingly, phase-matching is obtained for an almost identical width (≈ 1 µm), and the SHG efficiency expected from numerical simulations (not shown) is also very similar for the two devices: η = 300% W^−1^mm^−2^ (nanowire) and η = 401% W^−1^mm^−2^ (nanorib).

Propagation losses at ω and 2ω were measured by acquiring Fabry–Perot transmission interference fringes in on-purpose processed 200 µm long waveguides terminated by flat ICP etched facets (which have higher reflectivity than the tapered counterparts). The combined loss–reflection coefficient R’ = R exp(-αL) can be extracted by the contrast K of the transmission fringes as follows:

(1) $$K=\frac{T_{\max}-T_{\min}}{T_{\max}+T_{\min}}$$

(2) $$R\prime =\frac{1-\sqrt{1-K^{2}}}{K}$$

where L is the length of the waveguide and T_max_ and T_min_ are the maximum and minimum transmission values, respectively. The modal reflectivity R was calculated at both ω and 2ω via 3D FDTD modeling, and the propagation loss coefficient was then found as:

(3) $$\alpha =\frac{1}{L}\ln\left(\frac{R}{R\prime }\right)$$

The coupling efficiency κ of a waveguide terminated by inverted tapers, assumed to be equal at the input and at the output, was finally obtained by measuring its overall transmission and dividing it by the propagation loss exp(-αL):

(4) $$\kappa =\sqrt{\frac{T}{e^{-\alpha L}}}$$

The results of the above linear characterization are summarized in Table 1. For both designs, for L = 1 mm, the propagation loss at ω is quite limited (e^-αL^ ≈ 70%), while at 2ω it turned out to be one order of magnitude higher, due to the proximity between photon energy at 2ω and the forbidden band (740 nm) and to stronger scattering at the waveguide sidewalls at shorter wavelength. As for the input/output coupling, we estimate that the efficiency at ω for the rib waveguides can reach the same level as in nanowires after further optimization of design and processing. The low coupling efficiency at 2ω is to be ascribed to the multimode nature of the waveguide at this wavelength.

**Figure 4:**
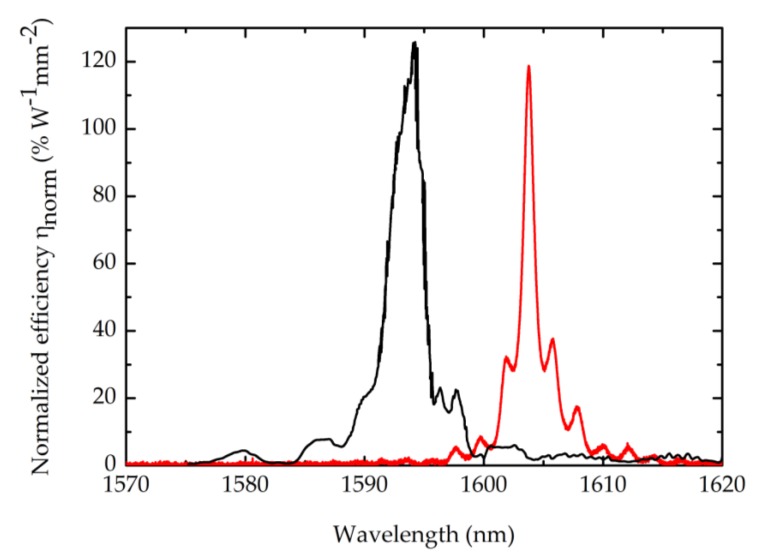
Nonlinear second-harmonic generation (SHG) efficiency spectra for the nanowire (black) and nanorib (red) waveguides.

Figure 4 shows the SHG efficiency spectra acquired by injecting and tuning the TE polarized telecom-range laser into a 1 mm nanowire (black trace) and a 200 µm long nanorib (red trace) waveguide, collecting the outcoupled TM mode at 2ω. The internal efficiency η was calculated by normalizing the overall efficiency P_SHG_/P_in_^2^ to the coupling efficiency at ω and 2ω:

(5) $$\eta =\frac{1}{\kappa_{\omega }^{2}\kappa_{2\omega }}\frac{P_{\mathrm{SHG}}}{P_{\mathrm{in}}^{2}}$$

with peak values of 16% W^−1^ (wire) and 3% W^−1^ (rib). The normalized efficiency equations ($\eta_{\mathrm{norm}}\;=\;\eta /L^{2}$) of the two devices are expected to be very similar; nevertheless, the ratio ($\eta_{\mathrm{wire}}/\eta_{\mathrm{rib}}$) does not scale as the square of the ratio of the lengths ${\left(L_{\mathrm{wire}}/L_{\mathrm{rib}}\right)}^{2}$. This is due to propagation loss at 2ω, which limits the interaction length to << $L_{\mathrm{wire}}$. By taking into account the effect of propagation loss on the efficiency η, we can calculate the normalized SHG efficiency $\eta_{\mathrm{norm}}$, defined as follows: [36]

(6) $$\eta =\eta_{\mathrm{norm}}L^{2}\exp\left[-\left(\alpha_{\omega }+\alpha_{2\omega }/2\right)L\right]\frac{{\sin h}^{2}\left[\left(\alpha_{\omega }-\alpha_{2\omega }/2\right)\frac{L}{2}\right]}{{\left[\left(\alpha_{\omega }-\alpha_{2\omega }/2\right)\frac{L}{2}\right]}^{2}}$$

obtaining 128% W^−1^mm^−2^ (wire) and 119% W^−1^mm^−2^ (rib). The results are summarized in Table 2.

## 4. Discussion

We demonstrated phase-matched optical SHG from the telecom range in suspended submicron AlGaAs waveguides with two different designs: a nanowire and a nanorib. The two approaches exhibit similar performances in terms of injection and propagation loss at ω, which are low enough to fabricate 1 mm long devices and in terms of nonlinear efficiency. Propagation loss at 2ω is intrinsically higher due to scattering and residual absorption, and it limits the SHG efficiency with respect to the expected values. Yet, the experimental conversion efficiency is higher than in oxidized form birefringent AlGaAs nonlinear waveguides (≈ 10% W^−1^mm^−2^), [37,38] and comparable to state-of-the-art SiO_2_ cladded submicron GaAs waveguides (≈ 130% W^−1^mm^−2^) [23]. While the optical performances are almost identical for the two designs, the nanorib exhibits far better mechanical properties. Its mechanical robustness makes its processing easier, not requiring CO_2_ supercritical drying, with a higher fabrication yield and less delicate handling. In addition, its ability to withstand several wetting and flash drying cycles without any damage makes the nanorib perfectly adapted to chemical and biological sensing in liquids, which could be easily injected through the etch windows. 

## Figures and Tables

**Table 1:** Measured linear optical features.

Design	λ (nm)	R (%)	L_trans_ (μm)	K (%)	Α (cm^−^^1^)	T (%)	κ (%)
Wire	1600	16.7	200	30.3 ± 0.3	3.7 ± 0.5	34.0 ± 0.2	60.7 ± 0.3
	800	24.3	200	23 ± 5	38 ± 12	0.50 ± 0.02	10.0 ± 1.0
Rib	1600	16.7	200	30.1 ± 0.6	4.0 ± 1.0	5.0 ± 0.2	23.3 ± 0.7
	800	24.3	200	22 ± 3	39 ± 7	0.50 ± 0.02	10.4 ± 0.7

**Table 2:** Measured nonlinear optical features.

Design	L_SHG_ (μm)	P_in_ (W)	P_SHG_ (W)	η (% W^−1^)	η_norm_ (% W^−1^ mm^−2^)
Wire	1000	8.0 × 10^−4^	3.9 × 10^−9^	16 ± 2	128 ± 20
Rib	200	4.0 × 10^−4^	2.7 × 10^−11^	3.0 ± 0.5	119 ± 20
